# 461. The Trifecta of Threat: National Burden of Infectious Disease Syndromes, Attributable Pathogens, and Antimicrobial Resistance Trends in India from 1990–2021

**DOI:** 10.1093/ofid/ofaf695.153

**Published:** 2026-01-11

**Authors:** Dhwani Vaghani, Sandeep Sekar Lakshmisai, Drumadala Gajbhiye, Chandana Tummala, Neelima Sinha, Thanmayee Tummala, Twisha Parikh, Sagar Patel, Chethan Raj Gundoji, Nikhil Rayarakula, Hardik Dineshbhai Desai

**Affiliations:** Internal Medicine, MGM Medical College, Aurangabad, Maharashtra, India,431003, Aurangabad, Maharashtra, India; Department of Medicine, SRM Prime Hospital, Chennai, Tamil Nadu, India, 600087, Chennai, Tamil Nadu, India; Government medical college Akola Maharashtra India 444001, Akola, Maharashtra, India; Internal Medicine, Vydehi Institute Of Medical Sciences and Research Centre, Whitefield-560066, Bangalore, Karnataka, India, Banglore, Karnataka, India; Department of Medicine, Kurji Holy Family Hospital, Patna, Bihar, India, 800010, Patna, Bihar, India; Bhaskar Medical College, yenkapally, Hyderabad, Telangana, India - 500075, Hyderabad, Telangana, India; GMERS Medical College and Hospital, Gotri, Vadodara, India 390021, Vadodara, Gujarat, India; Internal medicine -Ward Wizard Medicare Pvt Ltd, Nadiad, Gujarat, India, 387002, Nadiad, Gujarat, India; Internal Medicine, Government Medical College Nizamabad, Nizamabad, Telangana, India, 503001, Nizamabad, Telangana, India; College of Public Health, Kent State University, Kent,Ohio, USA, 44242., Kent, Ohio; Independent Researcher, Ahmedabad, Gujarat, India, 382350, Ahmedabad, Gujarat, India

## Abstract

**Background:**

Infectious diseases (ID) continue to pose a major public health challenge, with evolving pathogen profiles and growing antimicrobial resistance (AMR) in India. A syndrome-based approach integrating pathogen attribution and resistance trends offers vital insight into India's evolving infectious disease (ID) burden—yet long-term national data capturing these combined dynamics remains scarce.

**Figure:**
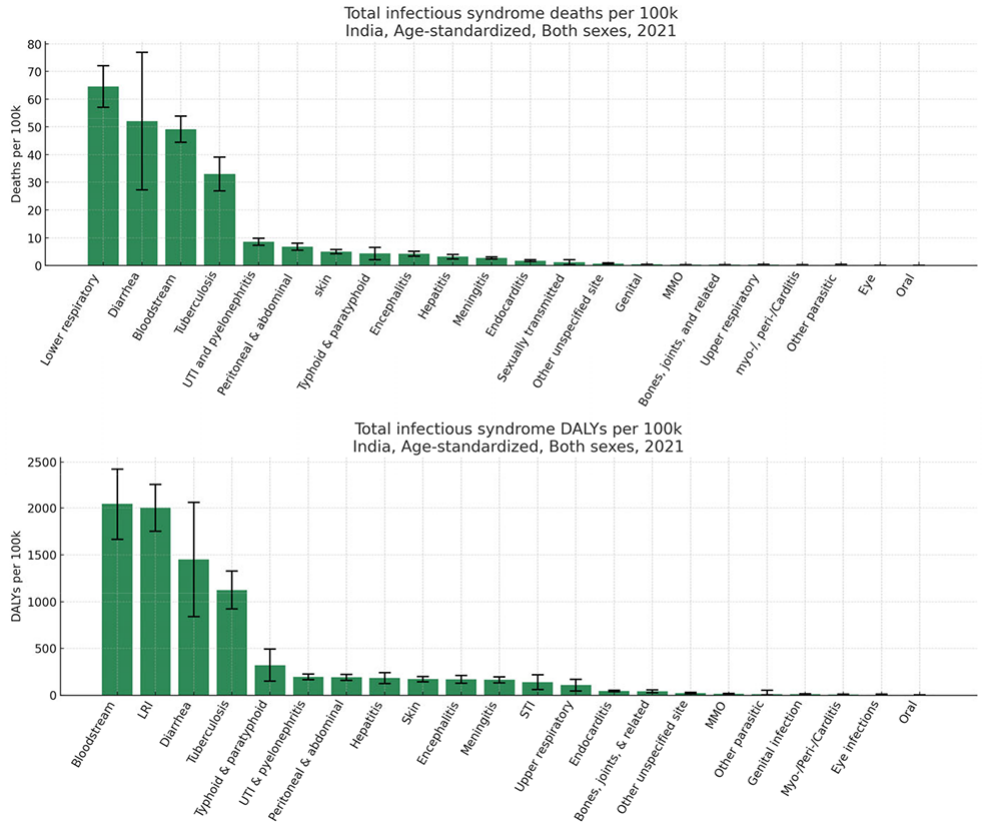
Burden by Infectious Syndrome in India, Both Sexes, Age-Standardized Rate (per 100,000), 2021

**Figure:**
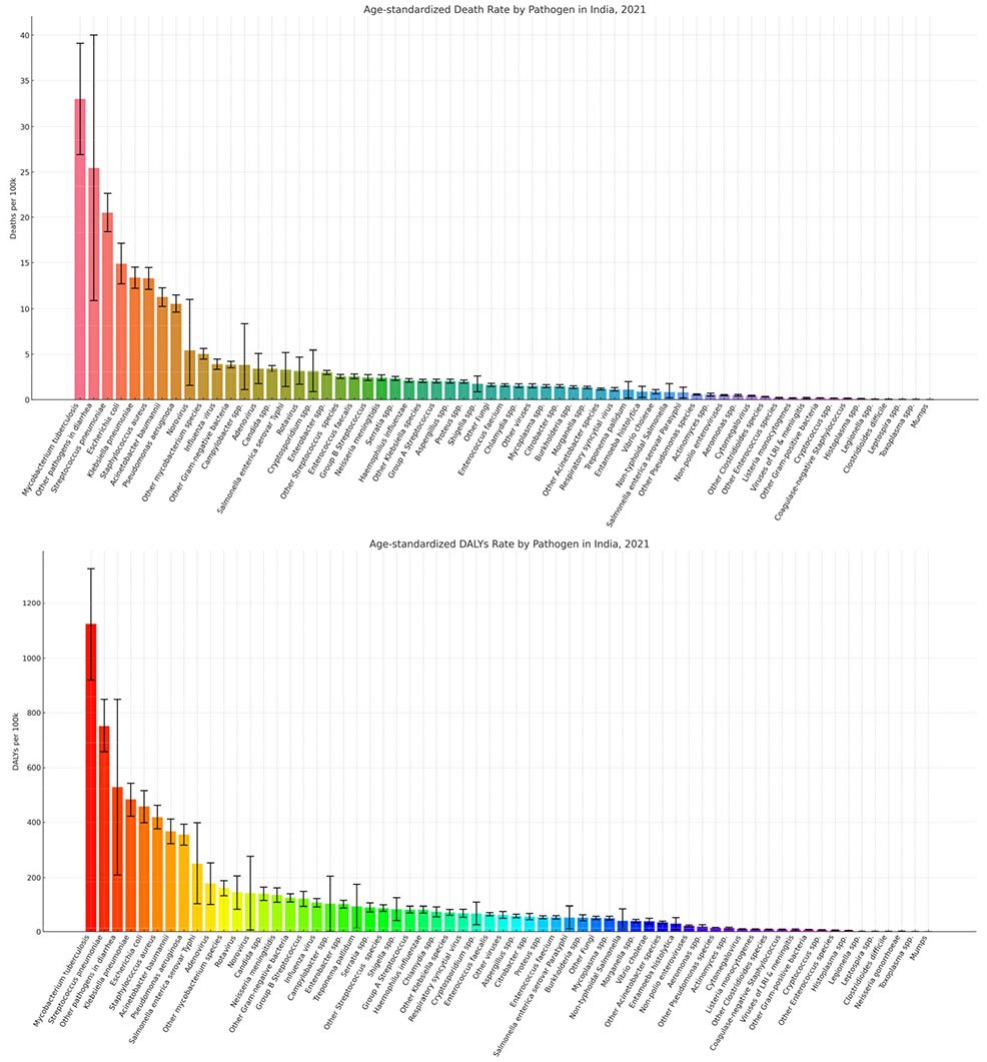
Burden by Pathogens in India, All Syndromes, Both Sexes, Age-Standardized Rate (per 100,000), 2021

**Methods:**

We performed a retrospective, population-based analysis covering the period 1990–2021 to estimate mortality and disability-adjusted life years (DALYs) across major ID syndromes in India using global burden of disease AMR study 2021. Resistance trends were evaluated by analyzing antimicrobial susceptibility patterns and their association with mortality and DALYs attributable to AMR.

**Figure:**
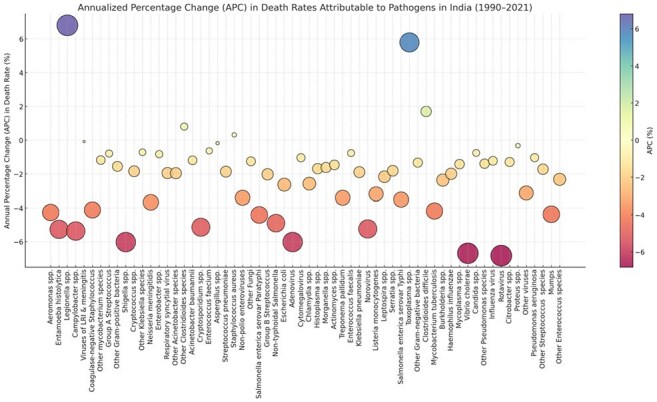
Percentage Change in Mortality Rate by Pathogen, All Infectious Syndrome, Annual Percentage of Change (APC) (%) from 1990-2021, Age-Standardized Rate per 100,000

**Figure:**
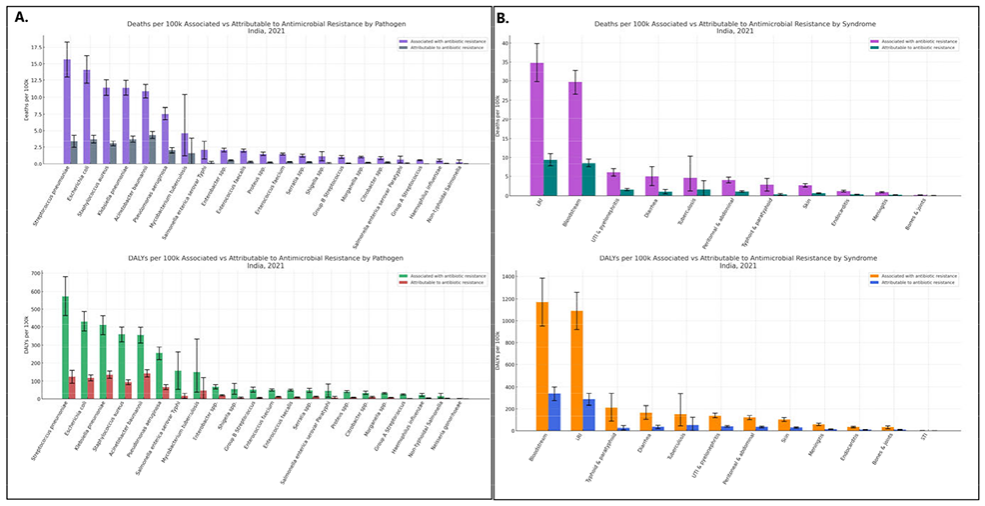
Attributable and Associated Antimicrobial Resistance by Pathogen and Infectious Syndrome in India, Age-Standardized Rate per 100,000, 2021

**Results:**

From 1990-2021, total deaths from all ID syndromes in India increased from 4.0 million (95% UI: 3.7–4.4) to 4.3 million (4.0–4.6). The highest annual percentage of change (APC) increases in death counts were observed for eye infections (18.7%), oral infections (7.8%), endocarditis (7.0%), infections of bones, joints, and related organs (5.8%), urinary tract infections and pyelonephritis (3.1%), and genital infections (1.7%). The greatest APC increases in deaths from 1990-2021 were observed for Legionella spp. (8.7%), Toxoplasma spp. (6.5%), and Clostridioides difficile (4.2%), indicating a rising burden of select bacterial and parasitic pathogens over time. In 2021, the highest number of deaths associated with AMR in India were linked to *Streptococcus pneumoniae* (163,129), *Escherichia coli* (142,310), and *Klebsiella pneumoniae* (121,623). For deaths directly attributable to AMR, *Acinetobacter baumannii* (47,586), *Klebsiella pneumoniae* (39,740), and *Escherichia coli* (37,600) were the leading contributors.

**Conclusion:**

AMR poses a critical threat in India, with disproportionate burdens driven by specific pathogens and syndromes. Our findings highlight bloodstream and respiratory infections as the dominant contributors, and gram-negative bacteria as leading culprits. These insights underscore the urgent need for precision-guided AMR interventions and data-driven policy action.

**Disclosures:**

All Authors: No reported disclosures

